# The impact of liver disease on mortality in cystic fibrosis - a systematic review protocol

**DOI:** 10.12688/hrbopenres.13065.3

**Published:** 2020-12-22

**Authors:** Ao Sasame, Lucy Connolly, Emer Fitzpatrick, Diarmuid Stokes, Billy Bourke, Marion Rowland

**Affiliations:** 1 School of Medicine, University College Dublin, Dublin, Ireland; 2 Children’s Health Ireland at Crumlin, Dublin, Ireland

**Keywords:** Cystic Fibrosis, Liver Disease, Mortality, Children, Adults

## Abstract

**Background** Cystic fibrosis (CF) is a multiorgan disease affecting the lungs pancreas and gastrointestinal tract. Pulmonary complications are the most common manifestation of the disease. Recent advances in the treatment of pulmonary complications have resulted in substantial improvement in life expectancy. Less than 10% of persons with CF (PWCF) develop liver disease (CFLD). There is conflicting evidence about impact of liver disease on mortality in CF, with evidence suggesting that CFLD contributes to increased mortality in CF, while other studies suggest that the impact on mortality is limited. Understanding the contribution of liver disease to mortality in CF is essential if further improvements in life expectancy are to be achieved.

**Objective:** To document the impact of liver disease on life expectancy for PWCF.

**Methods: **This systematic review will be conducted in compliance with the Preferred Reporting Items for Systematic Reviews and Meta-Analysis Protocols (PRISMA-P 2015). PubMed, Medline and Embase will be searched for English language publications (1949-2020). Studies reporting the outcome for CFLD will be included where the definition of CFLD is outlined clearly in a CF population. Studies with and without a comparator will be evaluated. Clinical trials of ursodeoxycholic acid will be excluded as well as organ transplantation outcome studies. We will examine all-cause and specific causes of mortality.We will include transplantation in our estimates of all-cause mortality. The Axis Risk of Bias Tool for Observational Studies will be used to evaluate the quality of studies. We will provide a narrative synthesis of our findings using tabular formats to highlight any impact of liver disease on mortality in CF.

**Conclusion: **It is anticipated that this review will bring clarity to the question of whether CFLD shortens life expectancy in PWCF and stimulate new approaches to the management of CFLD.

## Introduction

### Rationale

Cystic fibrosis (CF) is an autosomal recessive disease affecting the lungs, pancreas, gastrointestinal and biliary tract due to mutations in the *CFTR* gene ^1, 2^. Recent advances in the management of CF have resulted in substantial improvements in life expectancy for persons with CF (PWCF) ^3^. Today, children born with CF in Europe or the USA can expect to survive at least into their fifth decade ^4^. However, while the management of pulmonary complications of CF has greatly improved, complications such as cystic fibrosis liver disease (CFLD) contribute to increased morbidity and mortality in CF ^5– 8^.

CFLD is a complex form of liver disease, the pathophysiology of which is poorly understood. CFTR is expressed on the biliary epithelium, resulting in abnormalities in the composition, consistency and flow of bile with obstruction of small intrahepatic bile ducts ^9^. The damage, which is localized to the intrahepatic ducts, leads to portal tract fibrosis with well-preserved hepatic architecture. However, while historically it was considered that CFLD was as a result of damage caused by abnormalities in bile consistency and flow, more recent evidence suggests that non-cirrhotic portal hypertension may be the predominant pathophysiology of CFLD in some patients ^10^. Supporting the concept of non-cirrhotic portal hypertension is that portal hypertension is a relatively early feature of the disease in the presence of well-preserved synthetic function. This, in contrast to other forms of pediatric liver disease, makes early diagnosis of CFLD particularly problematic ^11^.

The prevalence of CFLD ranges from 10–30%, depending on the criteria used to define liver disease, but less than 10% of PWCF have CFLD with portal hypertension ^9, 12^. To date there is no definitive diagnostic investigation to confirm or rule out the presence of CFLD and clinical examination by an experienced hepatologist still remains the gold standard for the diagnosis of CFLD ^13^. While there are a number of diagnostic criteria, there is no coherent diagnostic algorithm for the diagnosis of CFLD. There is, however, an emerging consensus that the liver disease status of PWCF should be subdivided into three groups: (i) those with severe CFLD with portal hypertension; (ii) those with moderate or non-specific changes including biochemical and radiological evidence of liver disease, which do not fulfill standard criteria for portal hypertension; and (iii) those with no evidence of liver disease ^12, 14, 15^.

The onset of CFLD is usually in the first or second decade, with few reports of new onset liver disease in adult life ^9, 11– 13^. Once a diagnosis is established, there are no clinical indicators that predict disease progression in children or adults ^11^.

There is no treatment for liver disease in CF until end stage liver disease is well established, when liver transplant may be considered in certain circumstances.

There is ongoing debate as to whether CFLD shortens life expectancy in CF, with some authors suggesting that liver disease does not affect outcome ^16– 18^, while we and others have shown that CFLD is associated with a worse prognosis ^5, 8, 19^. If life expectancy for PWCF is to be further improved, clearly identifying risk factors for mortality that may be amenable to treatment is important.

No systematic review (SR) yet exists examining evidence that liver disease is a risk factor for mortality in CF ( Prospero International Prospective Register of Systematic Reviews, Cochrane Database of Systematic Reviews accessed May 2020). That the literature in the field is equivocal is understandable for many reasons. CF is a rare disease and less than 10% of PWCF will develop clinically significant liver disease. Therefore, large, well conducted epidemiological studies with adequate follow-up are difficult to conduct. The diagnosis of CFLD is not straightforward and relies on phenotypic characteristics of the disease rather than a conclusive diagnostic test and therefore, national CF registries have difficulty capturing the prevalence or outcome for CFLD. In fact, three recent studies using national registry data have all used different criteria to define CFLD, and have all struggled to accurately measure follow-up because it is impossible to pinpoint with any accuracy the onset of CFLD ^19– 21^. Compounding all of these factors is the complicated nature of multidisciplinary care in CF, the complexity of data capture and interpretation across the transition from paediatric to adult care. Adult reports of CFLD have focused on liver related mortality, and for the most part have concluded that CFLD is not a risk factor for mortality ^17, 18^. However, studies that have included adult and children and have examined all-cause mortality have demonstrated that CFLD is a risk factor for reduced life expectancy in CF ^5^.

We hope that this SR will bring clarity to the field and determine if liver disease in CF shortens life expectancy. We will compare (i) all-cause mortality (to include hepatic, pulmonary, non-CF related mortality combined with the number of reported transplantations (liver or lung) and (ii) specific causes of mortality (hepatic, pulmonary) in those with CFLD to those with CF but no evidence of liver disease for each study. We appreciate that there are significant weaknesses in many of the published studies in the field, and the heterogeneity in the published studies prevents any form of meta-analysis. However, we hope that by documenting the outcome of published research in a straightforward, tabular form, we will drive further collaborative research to improve clinical care pathways for PWCF who have liver disease.

The aim of this SR is to evaluate the impact of liver disease on mortality in cystic fibrosis. We will examine all-cause mortality rates as well as specific mortality rates for pulmonary, hepatic causes of death for persons with CFLD and compare them to mortality rates in those with CF but no evidence of liver disease (comparator group).

### Objectives

This SR will address the following question: What is the impact of liver disease on mortality in cystic fibrosis?

The proposed SR will address the following objectives:

i. Examine crude and population-based mortality rates for participants with CFLD.
ii. Determine if liver disease contributes to an excess mortality in those with CFLD.
iii. Identify risk factors that may influence the mortality for patients with CFLD such as age, gender, FEV1, height, weight BMI and diabetes.
iv. In studies which report on the outcome of CFLD we will document the number of persons with CFLD who receive a liver transplant, lung transplant, or liver and lung transplant and typo include the number of transplants in the final estimate of all-cause mortality (see section Outcome Page 5). Studies reporting outcome data for Transplant Registries will be excluded because the number of PWCF evaluated for liver transplant, listed for transplant or listed but not transplanted is rarely if ever reported.

There is no intervention examined. The secondary aim of this SR is to provide clarity for clinicians on the risks associated with liver disease in CF, and help inform clinical care pathways for children and adults with liver disease in CF.

## Methods

### Eligibility criteria

***Study design***. We will include studies examining the outcome for liver disease in CF, in adults or children or both. Observational studies, including cross-sectional, retrospective and prospective/cohort/follow-up studies in any clinical care setting for PWCF will be included. Included studies must provide an estimate of the prevalence of CFLD in the CF population from which the study sample is drawn. We will include studies which used the definition of CFLD proposed by NACFF ^1^ or Eurocare Guidelines ^2^ or studies that limit their study population to participants with clinical or radiological evidence of portal hypertension, i.e. clinically palpable spleen with or without low platelet count or an enlarged spleen on ultrasound (>2 SD above spleen size for age or >13 cm in those over 17 years of age). While the optimal comparison is PWCF with no evidence of liver disease, we will include data from studies which do not have a comparator group. Studies where CFLD is not the primary outcome of the study, but which report liver disease as a risk factor for mortality or report the number of participants who are classified as CFLD and die will be included. There is no restriction on the duration or type of follow-up studies included. Studies using CF-specific registry data will also be included if there are no violations of the exclusion criteria. In studies where only summary data are presented, we will seek further information from the corresponding author. We will include only English language publications, where full text of the article is accessible.

***Time frame***. Studies since the first reports of CFLD in 1949 until September 21st 2020 will be considered, but with the acknowledgement that the treatment of CF and the definition of CFLD has evolved since the first reports of CFLD. Older studies which do not provide information on mortality or which only report post mortem data will be excluded.

***Outcome***. The outcome for this study is mortality. We will define all-cause mortality as death from any cause including hepatic, pulmonary, other CF related causes, non-CF related causes and transplantation. We will combine transplantation data (liver or lung) with mortality data as transplantation represents the death of the patients own organ. We will subdivide all-cause mortality into hepatic pulmonary, other and transplantation. Outcome data from transplant registries (see above) will be excluded

***Exclusions***. It is a concern for many in the field that there is a limited number of publications on the outcome of CFLD. In addition, many studies are poorly designed or described, with short follow-up, or report liver related mortality as the only outcome. This review will seek to perform a systematic and comprehensive review in the field and therefore will not exclude studies because of less than optimal study design, small sample size or where only liver related outcomes are the primary aim of the study. We will however carefully evaluate the quality of the studies and report the risk of bias (see below).

Specifically, studies fulfilling any of the following criteria will be excluded:

- Studies where the definition/identification of liver disease in CF is vague and poorly described will be excluded.
- Studies that examine the outcome for multiple causes of liver disease (e.g. biliary atresia, metabolic storage disorders, viral hepatitis) in children or adults.
- Studies where the number of reported participants with CFLD is small (<5) will not be included.
- Randomized trials of ursodeoxycholic acid for the management of CFLD will be excluded.
- Studies of autopsy results will not be included unless they include a comprehensive report of the CF population with CFLD that gave rise to the autopsy report.
- Studies that describe the outcome of transplanted organs (liver, lung, lung and liver) based on transplant registry data, with no reference to the underlying population of persons with CFLD who did not receive a transplant will be excluded. Most of these transplant registry studies focus on the success of the transplanted organ rather than the outcome for the person with CFLD.
- All reviews including SRs will be excluded.

***Classification of CFLD***. The classification of CFLD is evolving and therefore we will include a range of classifications such as that described by Colombo *et al.* ^16^, the NACFF ^1^ and European Classifications ^2^ and more recent classifications used by Cipolli *et al.* ^3^ and Palls *et al.* ^4^ as well as other classifications where there is well described homogenous groups of research participants with only minor differences in terminology or inclusion criteria.

The following definitions of CFLD will be used to divide studies into 2 groups;

### Group 1 Studies

Based on the North American Cystic Fibrosis Foundation Guidelines and the European Guidelines which consists of 2 categories of liver disease (clinically significant cystic fibrosis liver disease (CFLD) and moderate or non-specific changes suggestive of LD (NSCFLD) Specific Criteria are:

**Cystic fibrosis liver disease (CFLD)**. Participants with CFLD have evidence of clinically significant liver disease based on clinical examination and/or radiological evidence of portal hypertension. Clinical evidence of CFLD is defined as: (a) palpable firm liver on clinical examination with or without splenomegaly; (b) in the absence of a firm liver, a clinically enlarged spleen, with or without hypersplenism, having ruled out of other causes of splenomegaly or portal hypertension. Radiological evidence of portal hypertension is defined as: (a) spleen size of +2 SDs above the mean for age; (b) spleen size greater than 13 cm in those over the age of 17 years. Histological evidence of CFLD is also included in this category, as well as evidence of oesophageal varices or venous gastropathy at endoscopy.

**Non-specific cystic fibrosis liver disease (NSCFLD)**. Participants with NSCFLD have some clinical radiological or biochemical liver abnormalities but do not meet the criteria for CFLD. Clinical characteristics include a soft palpable soft liver, which does not have the firm characteristic of CFLD. Radiological parameters include changes to the appearance of the liver on ultrasound but which do not meet criteria for portal hypertension. Biochemical evidence of liver disease include persistent abnormalities of liver biochemistry above the upper limit of normal.

**No liver disease (NoLD)**. Participants with NoLD have no clinical, radiological or biochemical abnormalities consistent with NSCFLD or CFLD and these constitute the comparator group.

### Group 2 Studies

Multilobular biliary cirrhosis associated with portal hypertension as defined by as splenomegaly based on clinical examination, ultrasound or magnetic resonance imaging and with or without laboratory evidence of hypersplenism.

### Information sources

Ao and DS (College Liaison Librarian for the College of Health and Agriculture), developed the literature search strategies using words related to cystic fibrosis liver disease and outcome, all-cause mortality and liver specific mortality. The following databases will be searched: PubMed, Embase and Web of Science from 1949 to a final search date in September 2020. The reference list of included studies will be screened for additional papers. Proceedings of the North American Cystic Fibrosis Conference and European Cystic Fibrosis Conference will be searched between 2000 and 2019 and any abstracts that have been published in full included. For recent manuscripts with restricted access, we will request a soft copy from the authors of the manuscript where feasible or crude data if possible. The literature search will be limited to English language publications where full texts are available. Case reports/series will be reviewed to determine if they meet the inclusion criteria. Research letters will be considered if they describe specific information on outcome of CFLD and are not published subsequently or previously as longer manuscripts. Reviews of any nature will not be included. We will circulate a bibliography of the included articles to the SR teams as well as to the CFLD clinical expert.

### Search strategy

No study design, or time frame will be imposed on the search. Due to resource limitations, only studies published in English will be included. PubMed, Embase and Web of Science will be searched. DS will oversee the specific search strategies. The search will be developed with input from the project team led by AS. The following search strategy terms will be used and adapted with relevant thesaurus terms for each database: ((“Cystic Fibrosis” OR “Mucoviscidosis” OR “Vaincre la Mucoviscidos”) AND (“Liver Disease” OR “Liver Dysfunction” OR “Hepatic Diseases” OR “Portal vein hypertension” OR “Portal hypertension” OR “Portal congestion” OR “Liver cirrhosis” OR “Hepatic cirrhosis” OR “Liver fibrosis” OR “Hepatic fibrosis” OR “Liver disorder” OR “Hepatic disorder” OR “Liver illness” OR “Liver failure” OR “Hepatic failure” OR “Cystic Fibrosis Liver Disease” OR “Cystic Fibrosis Associated Liver Disease” OR “Cystic Fibrosis-Associated Liver Disease” OR “Cystic fibrosis-related Liver disease” OR “Cystic fibrosis related liver disease” OR “CF-related Liver Disease” OR “CF-associated liver disease” OR “CFLD” OR “CFALD” OR “CFRLD)) AND (“Mortalit*” OR “Death” OR “Death Rate” OR “Survival Rate” OR “Survival Time” OR “Survival Probability” OR “Mean Survival” OR “Cumulative Survival” OR “Fatality” OR “Fatality Rate” OR “Case Fatality Rate” OR “Fatal Outcome” OR “Lethal Outcome”).

The search strategy will be validated to ensure that the strategy retrieves a high proportion of eligible studies (May 2020). The search will be updated toward the end of the review to ensure any recent publications are included in the review (September 2020). Both Cochrane and PROSPERO will be searched for ongoing or published SRs in the area of CFLD (May 2020).

### Study records

An electronic laboratory notebook will be used to manage the progress of the SR. It will be hosted on Google Drive (University College Dublin, UCD) where all authors will have access to the documentation.

AO and MR will be responsible for ensuring that any amendments to the protocol are documented appropriately as well as recorded in the electronic laboratory notebook. Scheduled meetings via Zoom or in person will take place on a two weekly basis and decisions recorded in the electronic lab book. If the protocol needs amendment, each amendment will be dated, and a new version of the protocol created (version and date), together with a description the issue that gave rise to the problem, the rationale for the change and the change/s made added to the protocol. Each numbered amendment and appear in the Study Records section of the Protocol.

***Data management***. Following a search of the electronic databases, the final number of studies from all three electronic databases will be imported into Endnote and duplicates (two copies of the same paper) removed, as well as reviews. The Endnote database of included studies will be imported into Rayyan QCRI. Rayyan is a free web-based application, developed by Qatar Computing Research Institute (QRCI) that supports screening of abstracts and titles using a process of semi-automation while incorporating a high level of usability. We will initially pilot and test the process of screening and ensure that all reviewers are interpreting the inclusion and exclusion criteria appropriately.

Rayyan will be used to screen titles and abstracts against inclusion and exclusion criteria. Rayyan allows both independent and collaborative screening of abstracts by reviewers as well as links to full text of the published papers. Outcome of the decisions in Rayyan will be tracked using the PRISMA flow chart, and the electronic laboratory notebook (AS).

***Selection process***. Once the data has been imported in Rayyan QRCI, the number of studies (alphabetical order) will be divided between the two groups of reviewers (group 1 AS, MR; group 2 LC, EF). This will be a staged process as follows. In Stage 1, study title and abstract will be screened against the inclusion and exclusion criteria and studies that do not meet the criteria will be excluded. The review of studies in Rayyan will be done independently by both members of each team in Stage 1. Any articles in conflict in Stage 1 will be reviewed as full text in Stage 2 and the conflict discussed and resolved by the team. If difficulties persist, the conflict will be resolved in discussion with the other team.

In Stage 2, full text articles of all potentially eligible papers ("Include" and ‘Maybe’ classification in Rayyan) will be independently reviewed by each member of the team (50% of studies for each team) against the eligibility criteria. If a study meets the inclusion criteria and none of the exclusion criteria it will be included in the review. Any studies in conflict will be reviewed with members of the other team and a consensus reached. We will record all decisions for excluded studies in Rayyan, in the electronic lab notebook.

To avoid double counting, publications from the same CF centre/s or national CF database that examine a similar population/cohort using a previously reported definition of CFLD and outcome will receive particular scrutiny. BB will facilitate discussions of possible double counting of publications and where double counting is highly likely, only one publication will be included in the tables. This scenario is likely with reports of follow-up at different time points, and only the longest follow-up study will be included in the tables. In the interest of fairness, the earlier study will be noted specifically in the exclusion section in our manuscript.

Hand searching of references of included studies (AO, LC) will take place after the initial studies for inclusion are selected, and any missing studies added to the database and assessed for inclusion as per above.

An account of all decisions made during this process will be documented in electronic format and the protocol updated if any deviation from the original protocol is necessary (AS).

***Data collection process***. Information will be extracted from eligible studies using a summary of evidence table V2 (see *Extended data* *DOI* https://doi.org/10.5281/zenodo.4032408) ^22^. The summary of evidence table has been developed for this SR with the involvement of all the reviewers and BB. Parameters included study design and analysis, time frame, definition of CFLD, baseline population characteristics and risk factors for mortality. The denominator in the included studies, in particular the denominator of the eligible population or the denominator for mortality rates clarified for us which studies would not meet inclusion criteria e.g. studies based on transplant registry data. We selected three studies that we felt were representative of the field and each reviewer evaluated the table for ease of completion, clarity, and understanding of the meaning and scope of variables to be collected. We also discussed the recording of missing or not reported data. Following two rounds of data extraction, discussion and some amendments, the final Summary of Evidence Table was adopted by the group. No evaluation of observer variation was undertaken.

Each reviewer will upload their completed tables onto the shared Google Drive. There is a specific focus on the definition of CFLD used in eligible studies (with a list of different definitions included, and assignment to one of 2 groups of studies (see above), the implications of the definition used for the risk of bias in the study, and the clarity with which information on the numerator, denominator, and time frame is described so the crude or standardised mortality rates can be obtained or estimated with confidence. We will also evaluate where possible the number of persons with CFLD receiving a transplant (liver, lung, lung and liver). In this evaluation we will not include any study that uses transplant databases as the source of their data.

Where data is presented as summary measures or there is important missing data in studies after 2005, we will attempt to make contact with the corresponding or senior authors by email (n = 2) to request, where possible, anonymous raw data. This is a small research community and our collaborator BB will facilitate the process of ensuring that all such requests are received by a senior author of the study.

### Data items

The summary of evidence table (see *Extended data* https://doi.org/10.5281/zenodo.4032408) ^22^ will be completed individually by AO, EF, MR. The summary of evidence table will include the following data extraction fields (definitions provided as necessary):

- Bibliographic information
- Aims (as stated in study)
- Study design and time frame of the study
- Study population (single institution, multiple institutions, national/regional, national registry data, combination of above.)
- Classification used for CFLD with a particular focus on the clarity of the application of any cited definition including Koh ^15^, Colombo ^16^, European ^12^ or North American classification ^14^. Other reported modifications of CFLD classification ^3, 4^. As outlined above we will divide publications into 2 groups based on the definition of CFLD used (Page 6), and the reviewer will determine which classification is used and abstract risk factors and mortality data accordingly..
- Classification of portal hypertension.
- Exclusion criteria used in the study, with particular emphasis on the handling of liver, lung or lung and liver transplantation.
- Identification of the size of population from which the study population is drawn and the number of participants included in the analysis will be extracted for each study.
- Demographic and baseline data reported including age, gender, genotype, height, weight, body mass index, pulmonary function, meconium ileus (MI), treatment with ursodeoxycholic acid and presence of CF related diabetes, and the criteria CFRD used to define CFRD.
- Outcome data. Raw data on the number of deaths combined with the number of transplants (liver or lung), the proportion to the population who die or are transplanted in the CFLD group and comparison group, the duration of follow-up, the cause of death.
- Death rates/proportions will be collected for all-cause mortality that is the combined death rate for liver, pulmonary or other causes of death and the number of transplants reported. Post transplant mortality will not be evaluated. Specific mortality rates for hepatic pulmonary other and transplants performed will be extracted.
- Crude and standardised mortality rates as presented in the study will be extracted and if possible, extrapolation of data to a standard rate will be attempted.
- Risk factors for mortality if reported will be extracted including age, gender, nutrition and pulmonary function, CFRD and abnormal laboratory parameters.

The summary of evidence table will be piloted for clarity and completeness. The table will be available for use in soft or hard copy, and the output computerised in Microsoft Excel for analysis. A synthesis of the findings will be carried out and if there is sufficient data available, or if there is a possibility of getting raw data from authors, a more detailed analysis will be conducted.

As this review spans over 70 years, reporting of research studies as well as the management of CF has changed dramatically. While consideration of this factor is important, missing and/or unclear information will be noted and reported in summary tables with specific focus on the lack of information on the CF population from which the cohort is drawn and how the comparator population is selected.

AO, EF, MR will be involved in data extraction and discrepancies resolved by consensus.

### Outcomes and prioritization

The primary outcome for this study is the mortality in persons with CFLD compared to PWCF with no evidence of liver disease. We know there is variability in how the data are reported in different studies. We will collect crude mortality rates (percentages) or rates as a function of follow-up (person years) for those with CFLD (according to the two groups of studies outlined previously). Other outcome measures considered include median survival age and age at death.

All-cause mortality will be the combined number of deaths and transplants (liver, lung, heart) because transplantation represents a failure of the native organ. We will also provide specific mortality rates for pulmonary, hepatic and other causes of death. We will extract data on the number of transplants reported but we will not examine mortality post transplantation, as this is beyond the scope of this SR. We will not consider factors related to the age of diagnosis of CFLD or survival/mortality after diagnosis of liver disease. We will only consider mortality in those who meet standard criteria for the diagnosis of CF (sweat chloride >60mEqv, two disease causing mutations in the CFTR gene, or one disease causing mutation in the presence of a classical clinical picture of CF).

Outcome data measured are hard end points in this review. Composite, soft or patient reported outcomes will not be considered. The language in the review will use the words mortality and outcome interchangeably.

### Risk of bias in individual studies

To assess the risk of bias within included studies we will use The critical Appraisal tool for Cross-Sectional Studies (AXIS) ^23^ (see *Extended data* https://doi.org/10.5281/zenodo.4032408) ^22^. Each paper’s risk of bias will be scored as ‘Yes’, ‘No’, ‘Don’t Know’ based on the study design, description of the population from which the sample is drawn, the comparator group, relevant classification of liver disease and description of risk factors and outcome measures used including duration of any follow-up. This will facilitate the needs of this particular review because we know that the risk of bias is high in most publications and it will help identify potential bias and improve transparency of the assessment between reviewers. Scoring will be done independently by AO, EF, MR following refresher training and piloting of the assessment on three studies selected at random and completed individually by all three reviewers. MR, and AS have previous experience of using the AXIS critical appraisal tool.

No study will be excluded because of the risk of bias; however, we will group studies in tabular form based on their risk of bias in terms of high, medium or low. We will consider identifying the reasons for our assessment of bias but further consideration of this is required when all studies have been assessed.

### Data synthesis

***Data synthesis***. Based on our evaluation of publications to date it is clear that the level of heterogeneity across the studies, in terms of definition of CFLD, age of participants, and duration of follow-up is such that it will only be possible to provide a systematic narrative synthesis. A systematic narrative synthesis will be performed with information provided in text and tabular form to summarise the findings of the review. This SR will seek to demonstrate in tabular form the proportion of participants in individual studies with CFLD who died (all-cause mortality and liver related mortality) compared to those with no evidence of liver disease and the excess mortality rate in those with CFLD as presented in papers or calculated from data provided. Mortality rates, together with absolute rate differences, will be presented. If possible, standardised mortality rates will also be presented. The authors will endeavour to present clearly and easily understood tables that highlight the excess burden of disease experienced by those with CFLD. All-cause mortality and liver related mortality will be clearly defined and differentiated.

### Confidence in the cumulative estimate

While we know that the quality of many of the studies in the field is not optimal, we feel that formally grading the quality of the evidence is fraught with difficulties. However, we hope that presentation of the data, while highlighting the limitations of the various studies, will have merit in drawing attention to the risk of liver disease in CF.

### Dissemination

We will follow a phased approach to dissemination

1. The findings of this SR we anticipate will have implications for persons with CFLD. We will discuss the findings of the SR with the Cystic Fibrosis Association of Ireland, and seek their support in minimising any undue distress caused to those with CFLD who do not fully appreciate the implications of having CFLD.
2. The National Clinical Programme for Cystic Fibrosis is the national body that determines the appropriate care pathways for CF in Ireland. They are aware that this SR is commencing and we will inform them of our findings and the implications of liver disease as a risk factor for mortality in CF. We hope that new clinical pathways or guidelines for the prevention and management of CFLD will be instituted nationally.
3. We will seek to have a manuscript published as an open access publication.

### Study status

We have revised the documentation for this SR including summary of evidence tables, risk of bias forms, refined our inclusion and exclusion criteria for studies, established a data management structure and implemented a search strategy. We are currently hand searching and searching references and conference proceedings which will be added to the database of included studies (August 2020). We will run the final search on September 21 ^st^ 2020. Summary of Evidence and Risk of Bias Analysis will be completed by 30 ^th^ October 2020.

## Data availability

### Underlying data

No underlying data are associated with this article.

### Extended data

Zenodo: The Impact of liver disease on mortality in Cystic Fibrosis-A systematic review protocol-data. https://doi.org/10.5281/zenodo.4032408 ^22^.

This project contains the following extended data:

- Risk of Bias Form V2 The Impact of liver disease on mortality in cystic fibrosis.pdf
- Summary of Findings Table V2 The impact of Liver Disease on Mortality in CF.pdf

### Reporting guidelines

PRISMA-P checklist for ‘The impact of liver disease on mortality in cystic fibrosis - a systematic review protocol’ https://doi.org/10.5281/zenodo.4032408 ^22^.

Data are available under the terms of the Creative Commons Attribution 4.0 International license (CC-BY 4.0).
