# Supplementary material for: The impact of liver disease on mortality in cystic fibrosis - a systematic review protocol
Source: HRB Open Res. 2020 Dec 22;3:44. Originally published 2020 Jul 16. [Version 3] doi: 10.12688/hrbopenres.13065.3 (PMC7713883; doi:10.12688/hrbopenres.13065.3)
Supplement: Peer review history

# Peer review: Reviewer response for version 3

**DOI:** 10.21956/hrbopenres.14345.r28561

**Published:** 2021-01-13

**Authors:** Chee Y Ooi

**Affiliations:** 1 Discipline of Paediatrics, School of Women's and Children's Health, University of New South Wales, Sydney, Australia

I am happy with the authors reply and have no further comments.

Is the study design appropriate for the research question?

Yes

Is the rationale for, and objectives of, the study clearly described?

Yes

Are sufficient details of the methods provided to allow replication by others?

Yes

Are the datasets clearly presented in a useable and accessible format?

Not applicable

Reviewer Expertise:

NA

I confirm that I have read this submission and believe that I have an appropriate level of expertise to confirm that it is of an acceptable scientific standard.

# Peer review: Reviewer response for version 2

**DOI:** 10.21956/hrbopenres.14276.r28075

**Published:** 2020-12-08

**Authors:** Henkjan Verkade, Sanne Duursma

**Affiliations:** 1 Department of Pediatric Gastroenterology/Hepatology,University Medical Centre Groningen, University of Groningen, Groningen, The Netherlands; 2 Department of Pediatric Gastroenterology/Hepatology, University Medical Centre Groningen, University of Groningen, Groningen, The Netherlands

We would like to thank Dr Rowland and colleagues for taking the time and effort to respond to our earlier review. Please find below our point-by-point reaction.

1. The authors state “Our focus is an unbiased application of our inclusion and exclusion criteria rather than the selection of a specific period of interest". We do not want to argue the chosen focus, but we still have the opinion that the inclusion of patient data before life expectancy of CF patients had exceeded childhood and before decent treatment strategies for portal hypertension (and its complications) had become available creates a major bias on the outcome, namely liver disease-related mortality in CF patients. The authors may not as much aim to decrease bias on the inclusion and exclusion criteria, but rather on the outcome, i.e. liver disease-related mortality in CF patients. We feel that the inclusion and exclusion criteria are not themselves the goal(s) of the effort, but “merely” chosen means to acquire relevant information on the outcome.
2. We agree with the adaptations which have taken away our earlier reservation on this point.
3. The further clarification of the definitions have improved the manuscript. In particular, the analysis of patients with “clinically significant CF Liver Disease” (according to the NACFS and European guidelines) seems to us most objective and therefore, most unbiased and relevant.
4. We agree with the adaptations which have taken away our earlier reservation on this point.

Is the study design appropriate for the research question?

No

Is the rationale for, and objectives of, the study clearly described?

Partly

Are sufficient details of the methods provided to allow replication by others?

No

Are the datasets clearly presented in a useable and accessible format?

Not applicable

Reviewer Expertise:

Pediatric gastroenterology/hepatology, genetic liver diseases.

We confirm that we have read this submission and believe that we have an appropriate level of expertise to confirm that it is of an acceptable scientific standard, however we have significant reservations, as outlined above.

# Response

**Published:** 2020-12-11

**Authors:** Marion Rowland

**Affiliations:** University College Dublin, Ireland

We very much appreciate the reviewers taking the time to provide further very helpful comments on this protocol for a systematic review. The following is our response to Point 1

1. We fully concur with Dr Duursma and Prof Verkade concerns that including older studies could bias our outcome metrics due to the change in clinical practice, diagnostic investigations, and the management of both CF and CFLD.  Having had the opportunity to appraise most of the literature we have further clarified the protocol to state that included studies must provide details of the prevalence of CFLD in the population on which the study is based (Methods Page 5 Line 11,12).  This removes many of the older publications which we feel are giving rise to the reviewers’ concerns. In addition, it also allows a better comparison of outcomes in different populations when the underlying prevalence of  CFLD is known. As suggested by Prof Debray we will further subdivide the Tables of Evidence according to date of publication so that the reader will be clearly able to see how the mortality of both CF and CFLD has changed since the 1980s. At this stage we are not in a position to provide definite information on the earliest studies included as the review process is still ongoing, but it is expected that no  study pre 1990 will be included. However, we do wish to reassure Dr Duursma and Prof Verkade that their concerns will be to the forefront when we are finalising the list of included studies.

# Peer review: Reviewer response for version 2

**DOI:** 10.21956/hrbopenres.14276.r28305

**Published:** 2020-12-02

**Authors:** Dominique Debray

**Affiliations:** 1 Pediatric Hepatology Unit, Reference center for Biliary Atresia and Cholestatic Genetic Diseases, APHP-Hôpital Necker, Paris, France

Dr Sasame *et al*, propose a systematic review of published studies between 1949 and 2020 in order to provide a comprehensive review of the outcome of Cystic Fibrosis (CF) liver disease, and appreciate the impact of liver disease on mortality in CF. They will review hepatic outcomes as well as liver related extrahepatic outcomes. Since it can be expected that in the near future the outcomes (liver and all cause mortality) will improve with the use of CFTR modulator therapies, this study is timely and may serve as a comparator for future studies in the next decade.

 As the authors acknowledge, this is the first systematic review to examine the impact of liver disease on mortality in CF. It has become clear that the prognosis of liver disease is related to the development of portal hypertension and related complications while liver failure is a late event. We also have learned during the past decade that portal hypertension may develop in patients without cirrhosis, as a result of obstructive portal venopathy.

 Although I agree that they should include all studies meeting their inclusion criteria, regardless of the publication date, I would suggest that they analyse the data according to eras, since management of portal hypertension has improved overtime with the advent of liver transplantation (in the 80’s), band ligation and TIPS (in the 90’s). They may demonstrate reduced liver related mortality rates over time (including decreased rates of liver transplantations) as well as a decrease in all-cause mortality.

 I do not have other comments. I am happy with the amendments that have been made.

Is the study design appropriate for the research question?

Yes

Is the rationale for, and objectives of, the study clearly described?

Yes

Are sufficient details of the methods provided to allow replication by others?

Yes

Are the datasets clearly presented in a useable and accessible format?

Not applicable

Reviewer Expertise:

Liver disease in cystic fibrosis, Pediatric chronic cholestatic disease, Pediatric liver transplantation.

I confirm that I have read this submission and believe that I have an appropriate level of expertise to confirm that it is of an acceptable scientific standard.

# Response

**Published:** 2020-12-11

**Authors:** Marion Rowland

**Affiliations:** University College Dublin, Ireland

We thank Prof Debray for her very helpful comments on this protocol. We very much agree that examining the change in outcome in mortality for CFLD since the 1980s will provide important information in the field, as well as providing a useful baseline against which to assess the impact of modulator therapies on CFLD.

# Peer review: Reviewer response for version 2

**DOI:** 10.21956/hrbopenres.14276.r28332

**Published:** 2020-11-23

**Authors:** Chee Y Ooi

**Affiliations:** 1 Discipline of Paediatrics, School of Women's and Children's Health, University of New South Wales, Sydney, Australia

This proposal is most welcomed in the Cystic Fibrosis community. An important question, that needs to be addressed via a Systematic Review. 

 I have no concerns or comments apart from these:

1. There is an emergence of literature that CFLD may be related to CF portal venopathy rather than the traditional end stage cirrhosis with portal hypertension. This needs to be considered.
2. I am unsure if "treatment" effects can be considered (e.g. urso).
3. Will other co-factors that may impact on mortality (e.g. CFRD) be considered? A recent registry based paper by Singh H, *et al*. (2020) ^1^ suggested higher risk of CFRD etc.

Is the study design appropriate for the research question?

Yes

Is the rationale for, and objectives of, the study clearly described?

Yes

Are sufficient details of the methods provided to allow replication by others?

Yes

Are the datasets clearly presented in a useable and accessible format?

Not applicable

Reviewer Expertise:

NA

I confirm that I have read this submission and believe that I have an appropriate level of expertise to confirm that it is of an acceptable scientific standard, however I have significant reservations, as outlined above.

## References

[1] : Cystic Fibrosis-related Liver Disease is Associated With Increased Disease Burden and Endocrine Comorbidities. *J Pediatr Gastroenterol Nutr*.70(6) : 10.1097/MPG.0000000000002694 796-800 10.1097/MPG.0000000000002694 32443033

# Response

**Published:** 2020-11-26

**Authors:** Marion Rowland

**Affiliations:** University College Dublin, Ireland

We are grateful to Prof Ooi for his very generous comments about our protocol for a Systematic Review.

1. We share Prof Ooi’s enthusiasm for the new  pathology model  in CFLD. We, like Prof Choi, hope that portal venopathy may explain many of the unique clinical features of CFLD. Unfortunately, none of  the studies in the literature to-date on portal venopathy meet the inclusion criteria for this SR.
2. As outlined in our  Summary of Evidence Table 10.5281/zenodo.3900095. we will to extract data on URSO from the included studies.
3. Similarly, we will extract data as per the Summary of Evidence Table 10.5281/zenodo.3900095  and if possible, examine the relationship between other risk factors for mortality  including  Cystic Fibrosis Related Diabetes (CFRD),  gender, age, pulmonary function  and genotype in those with  liver disease in CF.

# Response

**Published:** 2020-12-11

**Authors:** Marion Rowland

**Affiliations:** University College Dublin, Ireland

We are grateful to Prof Ooi for his very generous comments about our protocol for a Systematic Review.

1. We share Prof Ooi’s enthusiasm for the new  developments in the underlying pathophysiology of CFLD. We, like Prof Choi hope that portal venopathy may explain many of the unique clinical features of CFLD. Unfortunately, none of  the studies in the literature to-date on portal venopathy meet the inclusion criteria for this SR.
2. As outlined in our  Summary of Evidence Table 10.5281/zenodo.3900095. we will to extract data on URSO from included studies.
3. Similarly, we will extract data as per the Summary of Evidence Table 10.5281/zenodo.3900095  and if possible, examine the relationship between other risk factors for mortality  including  Cystic Fibrosis Related Diabetes (CFRD),  gender, age, pulmonary function  and genotype in those with  liver disease in CF.

# Peer review: Reviewer response for version 2

**DOI:** 10.21956/hrbopenres.14276.r28076

**Published:** 2020-10-26

**Authors:** Michael Wilschanski

**Affiliations:** 1 Pediatric Gastroenterology Unit, Hadassah Hebrew University Hospital, Jerusalem, Israel

I am happy with the authors reply and have no further comments.

Is the study design appropriate for the research question?

Partly

Is the rationale for, and objectives of, the study clearly described?

Yes

Are sufficient details of the methods provided to allow replication by others?

Yes

Are the datasets clearly presented in a useable and accessible format?

Yes

Reviewer Expertise:

Pediatric Gastroenterology with an interest in Pancreas Research.

I confirm that I have read this submission and believe that I have an appropriate level of expertise to confirm that it is of an acceptable scientific standard.

# Peer review: Reviewer response for version 1

**DOI:** 10.21956/hrbopenres.14164.r27692

**Published:** 2020-08-11

**Authors:** Henkjan Verkade, Sanne Duursma

**Affiliations:** 1 Department of Pediatric Gastroenterology/Hepatology,University Medical Centre Groningen, University of Groningen, Groningen, The Netherlands; 2 Department of Pediatric Gastroenterology/Hepatology, University Medical Centre Groningen, University of Groningen, Groningen, The Netherlands

The authors aim to provide clarity on the impact of liver disease on mortality in patients with cystic fibrosis, based on earlier published data. The authors propose a systematic review of English language publications between 1949 and 2020, in which liver related and all-cause mortality is reported. These reviewers have several reservations on the validity of the approach:

1. The range of 1949-2020 does not seem logical. The life expectancy of CF patients was still below 10 years in the sixties and only the last decade this has increased to 40 years and above. It does not seem appropriate to combine data of 1949-2020 in which era the treatment and prognosis of CF has changed so profoundly.
2. These reviewers consider it rather arguable that mortality can be derived from retrospective registration as liver-related mortality. Our recent study indicated an considerably increased mortality risk of CF patients with cirrhosis (Pals *et al*. (2019)) ^1^, but interestingly the herein reported primary cause of death was pulmonary in 68% of cases (!), and liver failure related only in 18% of cases. This intriguing observation could have several causes, such as that liver disease (cirrhosis) aggravates pulmonary CF disease, but could also be due to bias in reporting the cause of death, or still other explanations. The proposed outcome parameter ”liver-related mortality” could thus very well provide a significant underestimation of the impact of (severe) liver involvement on mortality in CF patients.
3. The proposed strategy lacks a clear definition of what is understood by liver disease in CF patients, but will use manuscripts with “an unambiguous definition of liver disease”. However, a definition that is unambiguous may not be comparable to another definition, despite that the latter may be unambiguous as well. It is well known that serum parameters on liver functions may fluctuate in CF patients and their relationship to (severe) liver involvement has not been defined. Comparing studies with different definitions seems hazardous. Perhaps only patients with “clinically significant liver disease” (NASPGHAN definition) seems therefore most justifiable for analysis, in contrast to the patients with non-specific cystic fibrosis liver disease (NSCFLD).
4. Although the reviewers do appreciate that transplant-related mortality is likely not relevant for the objective of the present study, the fact that a patient is liver-transplanted implies a decreased “survival with native liver” what could be regarded as an outcome and of an indication for the severity of the liver involvement. It does seems reasonable to assume that the need for transplantation does affect patient survival and therefore this should one way or another be accounted for, according to these reviewers.

Is the study design appropriate for the research question?

No

Is the rationale for, and objectives of, the study clearly described?

Partly

Are sufficient details of the methods provided to allow replication by others?

No

Are the datasets clearly presented in a useable and accessible format?

Not applicable

Reviewer Expertise:

Pediatric gastroenterology/hepatology, genetic liver diseases.

We confirm that we have read this submission and believe that we have an appropriate level of expertise to state that we do not consider it to be of an acceptable scientific standard, for reasons outlined above.

## References

[1] : Cirrhosis associated with decreased survival and a 10-year lower median age at death of cystic fibrosis patients in the Netherlands. *J Cyst Fibros*.18(3) : 10.1016/j.jcf.2018.11.009 385-389 10.1016/j.jcf.2018.11.009 30558881

# Response

**Published:** 2020-10-02

**Authors:** Marion Rowland

**Affiliations:** University College Dublin, Ireland

We are very grateful to the reviewers for their constructive comments on our protocol for a systematic review (SR). We apologies for any confusion in the protocol regarding the definitions, inclusion and exclusion criteria used for study selection and data extraction. The following are our specific responses to the reviewers very constructive comments, and we also have made some amendments to improve the protocol which are outlined in the update text.

We thank Dr Duursma and Professor Verkade for their very helpful comments, and we hope we have fully addressed their concerns in the revised protocol methodology.

1.    To our knowledge this is the first SR to examine the impact of liver disease on mortality in CF and not to include studies which meet our inclusion and exclusion criteria, regardless of publication date, would introduce significant selection bias to the SR. Our focus is an unbiased application of our inclusion and exclusion criteria rather than the selection of a specific period of interest. Furthermore, we would like to emphasise that we will not include post-mortem studies or descriptive studies of CFLD with no information of outcome (Page 6 Line 36-37). As we now have greater clarity as to the quality of the data available to us than when we drafted the protocol, a narrative synthesis is the only option to present the results for this SR. It will not be possible to combine data from different studies or different time periods  in a meta-analysis. We have amended this in Page 12 Data Synthesis.

2.    We agree with the reviewer that patients with CFLD rarely die from liver related causes, and we have amended to protocol to clarify the definitions mortality used for data extraction. (Page 5 Lines 2-5, 12-13,25-28; Page 6 Lines 14-19; Page 11 Lines 33-36 ). As pointed out by Reviewer 2 a transplanted organ represents a “decreased survival of the native liver” (Point 4)  and we very much concur with the statement. Therefore, we include transplants (liver, lung, or heart) in our estimates of all-cause mortality. The following is the outcome data that we will extract from the published literature (Summary of Evidence Table https://doi.org/10.5281/zenodo.4032408)

a.     All-cause Mortality (death from any cause and transplantation (lung or liver) regardless of post-transplant survival 

b.    Specific mortality rates 

i.    Liver related mortality, 

ii.    Pulmonary related mortality, 

iii.    Other causes of mortality

iv.    Transplant -number of transplants (liver and/or lung) reported in those with CFLD and the comparator group.

While studies reporting only liver related mortality will meet our inclusion criteria the lack of all-cause mortality data will be highlighted as we like the reviewer feel that this is important in understanding the impact of liver disease on mortality in CF. 

3.    Our aim is to provide a comprehensive review of the outcome of CFLD. We acknowledge that there are many opinions as to how best to classify CFLD1-6. We propose to broadly categorise the various definitions of CFLD reported in the literature into two groups as follows: (i) Group 1 those studies which include a “moderate” or “nonspecific” liver disease group as well as a severe liver disease group.1-3 and (ii) Group 2 those studies  which only examine severe liver disease with or without a comparison group4,5, We agree with the reviewer that our term “unambiguous definition of CFLD” has caused confusion and have rephrased this section in the abstract and the methodology (Page 6 Line 28-29). As the ESPGHAN definition of clinically significant liver disease has not been published to date, (personal communication) we have not identified any publication specifically referencing this classification among our included studies. 

4.    We fully agree that transplantation must be considered as a death of the native organ (lung or liver). Therefore, in our all-cause mortality rates we have defined all-cause mortality as death from any cause (pulmonary/hepatic/accidental/other)  combined with transplantation of single organ  (liver or lung) or multiple organs  (heart/liver/lung/pancreas/kidney)  as outlined in Point 2 (Page 6 Lines 14-19; Page 11 Lines 33-36) and our Summary of Evidence Table https://doi.org/10.5281/zenodo.4032408.

In the protocol we have used the terminology “transplant related mortality” which we have now removed.  Only the number of transplants will be included. Any evaluation of transplant related mortality is beyond the scope of this SR and have clarified this (Page 11 Lines 38-39)

5.    We trust that the above responses and the changes outlined below will give assurance to the Reviewer that the methodology is appropriate to address the question of the impact of liver disease on mortality in cystic fibrosis.

1.    Colombo C, Battezzati PM, Crosignani A, et al. Liver disease in cystic fibrosis: A prospective study on incidence, risk factors, and outcome. Hepatology 2002; 36(6): 1374-82.

2.    Debray D, Kelly D, Houwen R, Strandvik B, Colombo C. Best practice guidance for the diagnosis and management of cystic fibrosis-associated liver disease. J Cyst Fibros 2011; 10 Suppl 2: S29-36.

3.    Flass T, Narkewicz MR. Cirrhosis and other liver disease in cystic fibrosis. J Cyst Fibros 2013; 12(2): 116-24.

4.    Cipolli M, Fethney J, Waters D, et al. Occurrence, outcomes and predictors of portal hypertension in cystic fibrosis: A longitudinal prospective birth cohort study. Journal of Cystic Fibrosis 2109; 16.

5.    Pals FH, Verkade HJ, Gulmans VAM, et al. Cirrhosis associated with decreased survival and a 10-year lower median age at death of cystic fibrosis patients in the Netherlands. J Cyst Fibros 2019; 18(3): 385-9.

# Peer review: Reviewer response for version 1

**DOI:** 10.21956/hrbopenres.14164.r27690

**Published:** 2020-07-27

**Authors:** Michael Wilschanski

**Affiliations:** 1 Pediatric Gastroenterology Unit, Hadassah Hebrew University Hospital, Jerusalem, Israel

Dr Sasame and colleagues propose to conduct a systematic review of published studies since 1949 of liver-related mortality in Cystic Fibrosis. This group is possibly the number 1 center world-wide to perform such an analysis as they have been at the forefront of liver disease research in CF for decades with numerous seminal publications on the subject.

 This study is timely but I have 2 major suggestions.

 If the study is to study mortality the "group 2" patients with non-specific biochemical and even radiological evidence of liver disease should be left out. We do not know if any of this group of patients progress to life-threatening disease and the waxing and waning of blood tests could be part of the natural history of CF and have no effect long term at all. The study of this group will almost certainly blur the results. The study must concentrate on those persons with severe liver disease with portal hypertension. This leads me to my second criticism that studies describing transplanted patients should be included because this is just the population-that of severe disease-which needs to be included.

 I feel that Patient Registries should be accessed because they have the cohort of severe liver disease which this study is looking for.

 A minor criticism is looking back to 1949 which really is pre-history. I dread to think what the liver of CF patients were like then and there may be other variables which will blur the results. In the pre-PERT era severe liver disease could have been due to malnutrition which, hopefully, does not happen today. Could I suggest going back 30 years only?

Is the study design appropriate for the research question?

Partly

Is the rationale for, and objectives of, the study clearly described?

Yes

Are sufficient details of the methods provided to allow replication by others?

Yes

Are the datasets clearly presented in a useable and accessible format?

Yes

Reviewer Expertise:

Pediatric Gastroenterology with an interest in Pancreas Research.

I confirm that I have read this submission and believe that I have an appropriate level of expertise to confirm that it is of an acceptable scientific standard, however I have significant reservations, as outlined above.

# Response

**Published:** 2020-10-02

**Authors:** Marion Rowland

**Affiliations:** University College Dublin, Ireland

We are very grateful to the reviewers for their constructive comments on our protocol for a systematic review (SR).  We apologies for any confusion in the protocol regarding the definitions, inclusion and exclusion criteria used for study selection and data extraction. The following are our specific responses to the reviewers very constructive comments, and we also have made some amendments to improve the protocol which are outlined in the update text.

We thank Prof Wilschanski for his very generous acknowledgement of our work. 

1.    We agree with Prof Wilschanski that it would be a much simpler systematic review (SR) if we limited our inclusion criteria to only papers which reported severe CFLD with an appropriate comparison group. However, our aim is to provide as comprehensive review as possible about the impact of liver disease on mortality in CF. Because there are several different classifications of CFLD in the published literature ^1-6^ we have amended the protocol to better reflect the classification of liver disease as reported by individual studies. Specifically, we will divide studies into 2 groups: (i) Group 1 those studies which include a “moderate” or “nonspecific” liver disease group as well as a severe liver disease group. ^1-3^ and (ii) Group 2  those studies which only examine severe liver disease with or without a comparison group ^4,7^ 

We have amended the Protocol as follows: Page 3 Line 17  added “moderate” to describe the less severe group of CFLD; Page 5 moved Lines 38-41 into the Study Design Section; with further changes to Page 7 Lines 2-10, 29-32; and  Page 11 Lines 19-22 to reflect the new groups of included studies. We have also amended the Summary of Evidence Table and data dictionary to clarify this change. (Extended Data https://doi.org/10.5281/zenodo.4032408

2.    To address Professor Wilshanski’s specific concern about those in “group 2” blurring the results our current analysis plan in this narrative synthesis is to generate individual Tables for the two groups of studies outlined in Point 1 above. Based on more detailed work since this protocol was developed, we feel that the studies are too heterogenous to allow  any summary calculations or meta-analysis and we will rely on tabular data to highlight the impact of liver disease in CF (Page 12 Data Synthesis).

3.    To clarify - we will include CF patient registry publications where there is a definition of CFLD consistent with published literature, and which includes a denominator for the population studied as well as mortality data (Page 6 Lines 6-7). We have carefully reviewed transplant registry data publications and we have excluded such studies from this SR because their primary focus is transplant-related outcome and not outcome for CFLD. In general, these studies do not provide any data the number of patients with CFLD who were not evaluated for transplant, evaluated but not registered for transplant or listed for transplant but did not receive a transplant (Page 6 Lines 38-42).

4.    To our knowledge this is the first SR to examine the impact of liver disease on mortality in CF and not to include studies which meet our inclusion and exclusion criteria, regardless of publication date, would introduce significant selection bias to the SR. While we may demonstrate reduced mortality rates for CFLD over time our focus is an unbiased application of our inclusion and exclusion criteria rather than the selection of a specific period of interest.

**References**

1.    Debray D, Kelly D, Houwen R, Strandvik B, Colombo C. Best practice guidance for the diagnosis and management of cystic fibrosis-associated liver disease. J Cyst Fibros 2011; 10 Suppl 2: S29-36.

2.    Flass T, Narkewicz MR. Cirrhosis and other liver disease in cystic fibrosis. J Cyst Fibros 2013; 12(2): 116-24.

3.    Colombo C, Battezzati PM, Crosignani A, et al. Liver disease in cystic fibrosis: A prospective study on incidence, risk factors, and outcome. Hepatology 2002; 36(6): 1374-82.

4.    Cipolli M, Fethney J, Waters D, et al. Occurrence, outcomes and predictors of portal hypertension in cystic fibrosis: A longitudinal prospective birth cohort study. Journal of Cystic Fibrosis 2109; 16.

5.    Koh C, Sakiani S, Surana P, et al. Adult-onset cystic fibrosis liver disease: Diagnosis and characterization of an underappreciated entity. Hepatology 2017; 66(2): 591-601.

6.    Wilschanski M, Durie PR. Patterns of GI disease in adulthood associated with mutations in the CFTR gene. Gut 2007; 56(8): 1153-63.

7.    Pals FH, Verkade HJ, Gulmans VAM, et al. Cirrhosis associated with decreased survival and a 10-year lower median age at death of cystic fibrosis patients in the Netherlands. J Cyst Fibros 2019; 18(3): 385-9.
